# Is Quadriceps-Strengthening Exercises (QSE) in Medial-Compartment Knee Osteoarthritis with Neutral and Varus Malalignment a Paradox? – A Risk-Appraisal of Strength-Training on Disease Progression

**DOI:** 10.5704/MOJ.2403.010

**Published:** 2024-03

**Authors:** R Garg, A Krishna, R Daga, S Arora, S Puri, M Kumar

**Affiliations:** 1 Department of Orthopaedics, Maulana Azad Medical College, New Delhi, India; 2 Department of Radiodiagnosis, Maulana Azad Medical College, New Delhi, India; 3 Department of Radiodiagnosis, Govind Ballabh Pant Hospital, New Delhi, India

**Keywords:** knee osteoarthritis, medial compartment, quadriceps exercises, disease progression

## Abstract

**Introduction:**

The present inquiry seeks to investigate whether the current regimens of QSEs (Quadriceps-Strengthening Exercises) aggravate the disease while mitigating symptoms.

**Materials and methods:**

A comparative study was conducted on 32 patients with medial compartment osteoarthritis of knees. While the neutral group of 16 patients was constituted of those with an anatomical-lateral-femoro-tibial-angle (aFTA) 176-180º, varus group comprised an equal number of patients with an aFTA >180º. A home-based 12-week strength-training program involving weekly visits to hospital for supervised sessions was administered. The outcome measures were visual-analog-scale (VAS), medial patello-femoral joint tenderness (MPFJT), time-up-and-go-test (TUGT), stair-climb test, step test, WOMAC, IKDC scores, aFTA, hip-knee-ankle (HKA) angle, lateral-tibio-femoral-joint-separation (LTFJS), and horizontal-distance-from-centre-of-knee-to-Mikulicz-line.

**Results:**

There was a significant increase in quadriceps strength (p<0.01) in both groups. Values for neutral group with VAS score (p=0.01), MPFJT (p=0.01), TUGT (p=0.01), timing of the stair climb test (p=0.01), WOMAC (p<0.01), and IKDC (p=0.03) were better compared to varus group with VAS score (p=0.13), MPFJT (p=0.03), TUGT (p=0.90), timing of stair climb test (p=0.68), WOMAC (p<0.02), and IKDC (p=0.05). Varus group also showed an increase in aFTA and LTFJS in 12 patients, increase in HKA in 11, and increase in horizontal distance from the centre of knee to the Mikulicz line in 7 patients.

**Conclusion:**

The present study brings to the fore the paradoxical role played by QSEs in management of medial knee OA. While there is a radiological progression of the disease in both neutral and varus mal-aligned knees more so in the latter than the former.

## Introduction

Osteoarthritis of the medial compartment of the knee is a complex clinical entity resulting from a mutually aggravating interplay of biological and mechanical factors^1^. The mechanical axis of the lower limb (Mikulicz line) passes on an average 4 (±2) mm medial to the centre of the knee joint^2^. In a well-aligned knee, the load distribution is not uniform. Physiologically, 60% of the load is borne by the medial compartment while 40% is transmitted through the lateral compartment^3^.

Varus malalignment further increases the loading of the medial compartment by increasing the moment arm of the weight-bearing line^4^. These factors set in motion a cascade of changes leading to development of knee-pain and radiographic progression^4,5^. Knee-load during walking is also associated with subchondral bone changes, and cartilage loss^6-8^. Quadriceps strengthening exercises (QSEs) continue to hold the dominant position universally in the non-pharmaceutical remedies for osteoarthritis (OA) of knee joint^9-11^. However, a systematic review reported that only 56% of patients showed an improvement in the pain and functioning of the knee following strength training. Additionally, these benefits have not been reported to sustain beyond six months^10,12^. QSEs have not been found to be effective in reducing pain in patients with varus malalignment^13,14^. On the one hand, QSEs are necessitated by quadriceps wasting associated with OA^15^ but on the other hand, the surgeon has to reconcile with the fact that repetitive dynamic loading of knee mediates cartilage degeneration under the effect of axial compressive forces generated inside the joint by the cyclical quadriceps muscle contractions against resistance^16^.

Static knee malalignment is one of the most significant prognostic factors for disease progression in knee OA^14,17,18^. On the other hand, the effect of QSEs in enhancing muscle strength and improving pain and functioning of the arthritic knee has also been highlighted^13,19-21^. The role of QSEs in disease progression remains largely unanswered in the literature^10^. While confirming the paradoxical role QSEs play in management, our study throws up additional useful information and insight into the presently available literature on the relationship between QSEs and the radiological progression of disease.

## Materials and Methods

We prospectively performed the study in a tertiary-level referral teaching institute after clearance from institutional ethical committee. A written, informed consent was obtained from all the patients authorising radiological examination and photographic documentation. Patients presenting with medial compartment osteoarthritis between the age group of 40-70 years (fully ambulant with knee pain for 6 months or more without the aid of any walking devices) were recruited for the study. The patients with infective pathology, post-traumatic arthritis, focal chondral defects, degenerative subluxation of knee, ligamentous instability, and flexion deformity (>15°) or recurvatum (>5°) were excluded.

A total of 41 patients were initially recruited, of which 9 dropped out for not being able to make weekly hospital visits for supervised exercise sessions. This left 32 patients for analysis. The patients with lateral aFTA 176°-180° were included in the neutral group, and those with angle >180° in the varus group. In cases of bilateral knee pain with equal symptoms in both knees, the knee with greater pain or higher grading on the Kellgren-Lawrence (K-L) system was studied.

The patients were assessed at baseline and at the end of week 12. They were advised against any other interventions such as lateral-wedge insoles or intra-articular injections. The quadriceps strength was measured by a hand-held dynamometer (Precise Industrial Push-Pull Gauge). It graduates from 10N (1Kgf) to 50N (5Kgf). The patient was made to sit on a chair and strap was tied 5cm above the lateral-malleolus which was connected to the dynamometer (fixed to the undersurface of the chair). The patient was asked to extend the knee and the reading in the dynamometer was recorded.

Clinical outcome measures were visual analogue scale (VAS), medial patella-femoral joint tenderness, step test^13^ (it was used to assess dynamic standing balance. Participants were instructed to maintain a balance on the study leg while using the contralateral leg to step-on and -off a 15-cm-high step as many times as possible in 15 seconds without any weight transfer to the stepping leg), Time up and go test (TUGT) (the time taken to stand from a chair and walk 3 meters and then return and sit again), Stair climb test (time taken to ascend and descend a 6-step set of stairs), WOMAC, and IKDC scores.

Radiological outcome measures were measured manually: (1) HKA (Hip-Knee-Ankle) angle is the angle formed by the intersection of the mechanical axis of femur and tibia which is drawn on an ortho-scanogram; (2) aFTA is the lateral angle formed by intersection of lines between the anatomical axis of femur and tibia on an ortho-scanogram; (3) Lateral tibio-femoral joint separation (LTFJS) was measured directly as the joint space on bilateral knees, using Rosenberg view radiographs and was corrected for magnification based on the projected scale on the radiograph; (4) Horizontal distance from the centre of the knee to the Mikulicz line (i.e., the line connecting the centre of the hip to the centre of the ankle joint) was measured using an ortho-scanogram.

The quadriceps strength and outcome measures were recorded by one of the authors who was not privy to the allocation of the patients in the two different groups. After recording the baseline values of the outcome measures, patients in both groups were subjected to 12 weeks of QSEs. They were started with 20 repetitions of isometric exercises and 10 repetitions of isotonic exercises (without any weight on day one) with a muscle contraction for 10 sec. Thereafter, there was an increment of 2 repetitions in both exercise regimes every fortnight and an increase of 0.5kg of weight in isotonic exercises every month. The patients were instructed to follow the regime twice daily at their respective homes, and compliance was checked telephonically. The patients were followed-up in the hospital on a weekly basis for exercise sessions supervised by experienced musculoskeletal physiotherapists^13^.

The data was entered in MS Excel spreadsheet and analysis was done using Statistical Package for Social Sciences (SPSS) version 21.0 for Windows. Quantitative data was expressed in mean ± standard deviation format and the difference between the groups was tested by Mann Whitney ‘U’ test while pre-post data was compared by Wilcoxon sign rank test. Qualitative data was expressed in percentage and statistical differences between the proportions were tested by Chi-square test/ Fisher’s exact test. ‘P’ value of <0.05 was considered statistically significant.

## Results

The baseline-details of demographic, clinical and radiological parameters are given in Table I. Mean age was 47.75±3.08 years and 54.31±5.06 years in neutral and varus groups respectively. Among the 32 patients included in the study (16 in each group), 10 were males. Mean BMI was 23.50±2.30 and 23.77±2.53 in neutral and varus groups respectively. Nine out of 16 patients (56.2%) in neutral group belonged to K-L Grade 1, while the rest (43.8%) to Grade 2. Ten out of 16 patients (62.5%) in varus group belonged to KL Grade 3 while the other six (37.5%) to Grade 4.

**Table I:** Showing the demographic details, pain, functional and radiological outcome measures in patients belonging to both groups.

	**Neutral Knees (n=16)**			**Varus Knees (n=16)**		
Age (years)		47.75±3.08			54.31±5.06	
Male (%)		31.2			31.2	
Female (%)		68.8			68.8	
Symptoms U/L (n)		7				
B/L		9			16	
BMI (kg/m^2^)		23.50±2.30			23.77±2.53	
K/L Grade 1 (%)		56.2			--	
K/L Grade 2 (%)		43.8			--	
K/L Grade 3 (%)		--			62.5	
K/L Grade 4 (%)		--			37.5	
	**Baseline**	**Follow-up**	**p-value**	**Baseline**	**Follow-up**	**p-value**
Quadriceps Strength (N)	33.63±8.24	38.50±8.24	<0.01	25.25±7.18	30.94±8.21	<0.01
VAS	5.63±1.20	4.38±0.72	<0.01	5.88±0.72	5.56±0.72	0.13
Medial Patello-Femoral Joint Tenderness (MPFJT)	5.5±0.89	4.9±0.68	0.01	6.1±0.61	5.6±1.09	0.03
TUGT (sec)	15.13±2.53	13.44±2.45	<0.01	13.88±4.06	14.13±4.63	0.90
Stair Climb Test (sec)	19.50±5.11	18.19±4.79	0.01	18.88±2.82	18.06±4.72	0.68
Step Test (n)	17±3	18±3	0.18	18±4	19±4	0.45
WOMAC	49.13±21.28	41.88±19.23	<0.01	51.88±11.17	48.50±10.20	0.02
IKDC	33.50±15.23	35.63±14.58	0.03	29.38±13.88	32.88±14.57	0.05
aFTA (deg.)	177.50±1.15	178.38±2.16	0.02	182.81±1.56	184.19±2.10	<0.001
HKA Angle (deg.)	5.94±0.85	6.69±1.70	0.02	8.81±0.98	9.94±1.06	<0.001
LTF Joint Separation (LTFJS) (mm)	5.41±1.01	5.53±1.10	0.04	7.41±1.42	7.97±1.47	<0.01
Horizontal Distance from Center of Knee to Mikulicz Line (mm)	1.57±0.42	1.78±0.61	0.06	2.71±0.48	2.87±0.44	0.07

There was significant increase in the quadriceps strength (within group p<0.01; between group p=0.03) in both the groups. It increased from a baseline value (N) of 33.63±8.24 to 38.50±8.24 in neutral group and from 25.25±7.18 to 30.94±8.21 in varus group. QSEs led to an improvement in the knee pain and function in both the groups but the effects were significantly greater in neutral group (Tables I-III). Patients in neutral group reported a significant reduction in pain and tenderness as measured on VAS (p<0.01) and MPFJT (p=0.01) as compared to varus group (p=0.13) and (p=0.03) respectively. The neutral group patients reported a statistically significant reduction in the timing of TUGT (p<0.01) and stair climb test (p=0.01) as compared to the timing of TUGT (p=0.90) and stair climb test (p=0.68) in varus group (Table III). The mean number of steps in step test increased from 17 to 18 in neutral group, and from 18 to 19 in varus group, but this improvement was not statistically significant in either group. WOMAC and IKDC showed a significant improvement in both the groups with p<0.01 and 0.03 in neutral group and p<0.02 and 0.05 in varus group. The comparison of clinical and functional scores after the intervention across the two groups showed variable results. These values were VAS p<0.01, MPFJT 0.02, TUGT 0.92, step test 0.26, stair climb test 0.99, WOMAC 0.25, and IKDC 0.48) (Table II).

**Table II:** Showing comparison of clinical and radiological outcome measures between two groups pre- and post-intervention.

	Pre-intervention	Post-intervention
Quadriceps Strength (N)	0.01	0.03
VAS	0.63	<0.01
Medial Patello-Femoral Joint Tenderness (MPFJT)	0.03	0.02
TUGT (sec)	0.17	0.92
Stair Climb Test (sec)	0.32	0.99
Step Test (n)	0.20	0.26
WOMAC	0.79	0.25
IKDC	0.28	0.48
aFTA (deg.)	<0.001	<0.001
HKA Angle (deg.)	<0.001	<0.001
LTF Joint Separation (LTFJS) (mm)	<0.001	<0.001
Horizontal Distance from Center of Knee to Mikulicz Line (mm)	<0.001	<0.001

**Table III:** Symptomatic relief and radiological progression of disease after 12 weeks of QSEs.

	Neutral Knees (n=16)			Varus Knees (n=16)		
Pain and Functional parameters	Improved (n)/mean	Unchanged (n)/mean	Deteriorated (n)/mean	Improved (n)/mean	Unchanged (n)/mean	Deteriorated (n)/mean
VAS	10/4.0	4/4.75	2/5.5	8/6.13	5/5.6	3/6.3
Medial Patello-Femoral Joint Tenderness (MPFJT)	9/4.22	6/5.16	1/5	8/4.75	5/6.2	3/6.67
TUGT	12/12.83	1/14	3/16.33	8/12.25	2/11.5	6/17.33
Stair Climb Test	10/18.9	4/17	2/17.5	7/14.4	4/18.5	5/22.8
Step Test	7/18.7	5/18	4/18	9/20.44	2/16	5/17.8
WOMAC	12/41.25	4/42	0/0.00	11/49.54	4/43.5	1/48
IKDC	11/32.09	4/45.5	1/37	9/31.78	4/42	3/24
**Radiological parameters**	**Number of Patients with Radiological Progression of Disease (N)/Mean**					
aFTA		6/180.5			12/184.91	
HKA		6/8.3			11/10.18	
LTF Joint Separation (LTFJS)		4/6.5			12/8.08	
Horizontal Distance from Center of Knee to Mikulicz Line		4/2.56			7/2.9	

**Fig 1:**
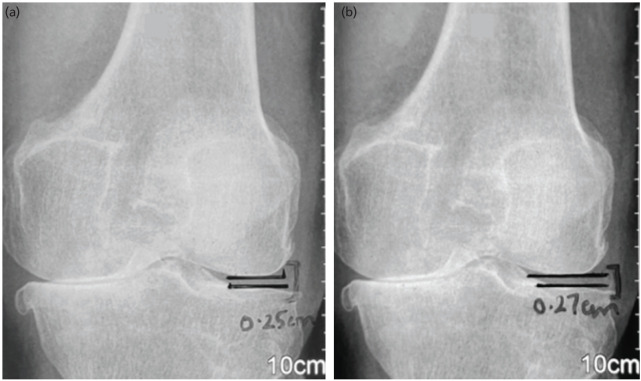
(a) Anteroposterior radiograph of the knee of a 63-years-old female (belonging to varus group) showing progression of lateral tibio-femoral joint space from pre-intervention radiograph to (b) post-intervention radiograph.

Despite showing an improvement in pain and functional outcome measures in both the groups, patients showed a radiological progression after 12 weeks of twice-daily QSEs. Twelve out of 16 patients in varus group as compared to only 6 out of 16 patients in neutral group showed an increase in aFTA (p=0.03). Similarly, 11 out of 16 patients in varus group as compared to 6 out of 16 patients in neutral group showed an increase in HKA (p=0.07). As for LTF joint space, it increased in 12 out of 16 patients in varus group (Fig. 1) as compared to only 4 out of 16 patients in neutral group (p=0.01). Also, the horizontal distance from the knee centre to Mikulicz line increased in 7 out of 16 patients in varus knees as compared to 4 out of 16 in neutral group (p=0.001) (Table I, III). The comparison of radiological outcome measures after intervention between the groups showed the p-values as: aFTA<0.001, HKA<0.01, LTFJS <0.001, horizontal distance from centre of knee to Mikulicz line<0.001 (Table II).

## Discussion

The present study provides a peek into the phenomenon of cartilage degeneration under the effect of current modes of QSEs over a short-term period of 12 weeks. The results obtained are in consonance with the long-term effects recorded in the literature^15,22^. The question regarding QSEs as a possible risk factor in disease progression and their paradoxical role in the management of medial knee OA has its origin in the common shared experience of the practitioners and researchers alike, namely, that although the current regimens of quadriceps strength training improve the knee functionality, the latter continue to progress towards arthroplasty. The articles by Bennell *et al*^6^, Sharma *et al*^14^, and Mikesky *et al*^15^ have made significant contributions to our understanding of this phenomenon. They complement our understanding of the short-term benefits of QSEs by providing a long-term perspective on the eventual outcome of strength training. There is a paucity of research on correlation between QSEs and disease progression (Table IV).

**Table IV:** Summary of reports studying the relationship of QSEs with clinical and radiological outcome measures.

S. No.	Author (year)	Country of origin	Methodology	Conclusion of the study	Comments by authors of the present study
Studies Comparing the Clinical Outcome Measures					
1.	Maurer *et al*^23^ (1999)	Country of origin	113 patients of knee osteoarthritis were divided into two groups: Gr 1 receiving 8 weeks of QSE; Gr 2 receiving health education lectures Outcome Variables: quadriceps strength, pain and functional measures	Both groups gained significant quadriceps strength Group 1 had more improvement in pain and functional outcome measures than group 2	The study makes no mention about the progression of disease. The study did not include the radiological parameters.
2.	Baker *et al*^9^ (2001)	USA	46 patients of knee osteoarthritis were randomized to a 4 month home based quadriceps strengthening training program	Home based strength training improved functions and quality of life in patients with knee osteoarthritis.	The study makes no mention about the progression of disease. Radiological parameters not used to study the relationship between quadriceps strength and knee OA
3.	Lim *et al*^13^ (2008)	Australia	Participants (n=173) were stratified into neutral and varus malaligned groups. Each group is then randomized into either a 12-week supervised home-based quadriceps strengthening group or a control group with no intervention. Primary outcome measure: knee adduction moment. Secondary outcome measures: step test, stair climb test, WOMAC score, isometric quadriceps strength	Quadriceps strengthening Had no effect on knee adduction moment in either group No improvement in functional outcome measures in either group Improvement in pain was seen in knees belonging to neutral group only	The patients in neutral and varus groups who were subjected to QSEs were found to have increased strengths post intervention. Radiological parameters not employed. No mention about the progression of the disease. Differential response to QSEs – relief in pain but no improvement in functions.
4.	Trans *et al*^21^ (2009)	Denmark	52 patients of knee osteoarthritis were divided into three groups: (1) Whole body vibration exercise (WBV) on a stable platform; (2) WBV-exercise on a balanced board; (3) Control group- no exercise. Group 1 and group 2 were trained for 8 weeks Outcome measures were – quadriceps strength, proprioception and WOMAC score.	The exercise regime improved strength and proprioception in group 1 and 2 but no improvement was seen in WOMAC score	Pain not included as an outcome measure. No improvement in functions. The exercises led to increase in strength Radiological parameters not employed to assess the progression of disease
5.	McQuade *et al*^20^ (2011)	USA	21 patients were assessed for pain and functional outcome measures after a supervised training program 3 times a weeks for 8 weeks.	Strength training reduced pain improved function	No mention about the progression of disease. The study did not include radiological parameters.
6.	Bennell *et al*^24^ (2014)	Australia	100 patients of knee osteoarthritis with varus malalignment were divided into two groups on the basis of exercise regime (neuromuscular exercise and quadriceps strengthening exercise) for 12 weeks. Primary outcome measure: knee adduction moment. Secondary outcome measure: WOMAC score and VAS score.	Strengthening exercises had no effect on knee adduction moment. Comparable improvement was seen in secondary outcome measures in both the groups.	Strengthening exercises improved functional outcome in malaligned knees. Strength training increased the quadriceps strength. No mention of radiological parameters.
Studies Comparing the Radiological outcome Measures					
7.	Ettinger *et al*^19^ (1997)	USA	439 patients were randomised into 3 groups: Gr 1 aerobic exercise training group; Gr 2 resistance exercise training group; Gr 3 health education group Outcome measures: (1) self-reported disability score; (2) knee pain score; (3) physical function; (4) radiograph score; (5) aerobic capacity; (5) knee muscle strength	After 18 months the participants in group 1 and 2 had improvements in disability score and pain score and had increase in muscle strength. But, there was no difference in radiographs scores between the exercise and health education group	The radiological parameter used in this study did not evaluated the progression of disease.
8.	Sharma *et al*^22^ (2003)	USA	237 patients divided into neutral and malaligned group which were further divided within the group on the basis of high and low quadriceps strength at baseline. The patients were followed for 18 months.	Greater quadriceps strength at baseline is associated with higher radiological progression of disease in malaligned knees	Radiological parameters used to assess the progression of disease. No strength training program was used; instead, the patients were prospectively followed to assess the progression of disease on the basis of baseline quadriceps strength. Complements the present study by providing a long-term effect of stronger quads on disease progression.
9.	Mikesky *et al*^15^ (2006)	USA	212 patients of medial knee osteoarthritis were stratified into knees without OA (K/L Grade 0,1) and knees with OA (K/L Grade 2-3). Each group was further stratified into strength training and ROM exercise group.	After 30 months of intervention: The rate of joint space narrowing in knees with OA (K/L 2,3) in strength training group at baseline was 37% slower than that in the ROM group. However, in patients without knee OA (K/L 0,1) instances of joint space narrowing exceeding 0.50mm were 79% more common in the strength group than in the ROM group	Radiological progression of disease was assessed. Similar to our study, this study also reported radiological progression of disease in knees with K/L 0,1 after strength training. In contrast to our study, the progression of disease was less after strength training in more osteoarthritic knees. Complements the work of the present study by providing the long-term profile of QSEs on disease progression.
10.	Present study (2021)	India	32 patients were divided into 2 groups on the basis of aFTA into neutral and varus malalignment. Both groups were subjected to 12 weeks of home-based quadriceps strengthening exercises supervised weekly. Pain and Functional outcome measures: Vas score Medial patella-femoral joint tenderness Step up and go test Stair climb test Time up and go test WOMAC score IKDC score Radiological outcome measures: aFTA HKA LTFJS Horizontal distance from center of knee to Mikulicz line	Quads strength is gained in both the neutral and varus knees. Improvement in pain and functional outcome metrics observed in both the groups with better response in neutral group. Close to 50% of patients either did not benefit or deteriorated on VAS and MPFJT scores. Radiological progression of disease observed in both neutral and varus knees under the effect of QSEs with higher deterioration in latter group. Closer is the knee varus to 10°, poorer are the VAS and MPFJT indices. Current regimens of QSEs be either suspended or modified if pain worsens on QSEs.	Results of the present study and review of literature on the relationship between QSEs and radiological progression of disease indicate that the subject can brook no further delay and calls upon the researchers and practitioners in the field to engage into multicentric long-term larger studies to examine and validate all the nuances of the phenomenon and identify alternate safer regimens of QSEs in the management of medial knee OA.

Sharma *et al*^22^ evaluated the correlation of quadriceps strength with progression of the disease in malaligned and lax knees over a period of 18 months and concluded that greater quadriceps strength at the baseline was associated with faster radiological progression of OA and that this relation was not observed in neutrally-aligned knees. However, the patients in their study were not subjected to any strength training program. Mikesky *et al*^15^ evaluated the effects of strengthening exercises on radiographic progression of disease in knees with K-L Grade >2 and <2. They did so without making any distinction between knees with varus and neutral alignments. They concluded that 30 months of QSEs led to a greater narrowing of the medial space joint in patients with radiologically normal knees. Our study plugs the gaps in these two long-term studies by prospectively examining the effect of QSEs in both the varus and neutral knees. However, the common thread that runs through these studies and the present work is that there is a radiological progression of disease under the effect of QSEs across both groups of knees even though this is accentuated 3-4 times^25-27^ in case of varus as compared to neutrally-aligned knee. Our study confirms the trend observed in the earlier studies regarding the progression of disease even in neutral knees.

An improvement in pain following QSEs in knee OA is widely reported in the literature, even though the mechanism behind this improvement is poorly understood^28-31^. However, it must also be borne in mind that around 50-60% of the patients either do not benefit from QSEs or have pain worse than before^12^. In the present study, 6 (out of 16) and 8 (out of 16) patients in neutral and varus knees respectively either did not show any improvement or deteriorated on their VAS and MPFJT. One plausible explanation for increased pain and patellofemoral joint tenderness available in the literature is that repeated quadriceps contractions against resistance in the presence of varus malalignment led to increased patellofemoral compression^32,33^. Therefore, it is imperative for the surgeon to understand that the nature of the exercises may directly predispose malaligned knees to greater patellofemoral joint compression, thereby, inducing increased knee pain.

There exists some degree of dissonance in the literature on the effects of QSEs on muscle strength and an improvement in the pain and functionality of the arthritic knee. Lim *et al*^13^ showed that while there was an increase in quadriceps strength after 12 weeks of strengthening program which relieved pain in the neutral group better as compared to the varus group. However, there was no improvement in functional outcome measures in either group. Trans *et al*^21^ showed that an increase in quadriceps strength after strength training was not associated with an improvement in the pain. Bennell *et al*^24^ showed that strength training increased quadriceps strength and reduced pain but did not investigate the correlation between the two.

Although the adduction moment, considered one of the biomarkers of disease progression, is not affected by QSEs^13,24^, it possibly generates sufficient axial compressive forces inside the joint to cause progression of disease by virtue of the fact that the quadriceps muscle spans the knee joint^16,34^. It is possible that overall compressive forces acting across the knee may increase with a quadriceps strengthening program, and this may have deleterious consequences for disease progression^13^. The exacerbation of knee pain in this group could possibly be attributed to the altered line of action of the quadriceps force in malaligned knees causing increased compressive forces on localised areas of articular cartilage^22,35^.

Although both the groups benefitted in our study, the beneficial impact was greater among the patients belonging to neutral group. While the improvement in functional outcome measures in both groups is in agreement with the observations reported elsewhere. The fact, that patients with Grade 4 disease in the varus group of our study too gained strength, runs counter to what has been reported in other studies. McQuade *et al*^20^ showed that while strength training may reduce pain, it may not necessarily increase quadriceps strength.

The difficulties faced in sustaining the strength training program in the present study were twofold. First, patients who began to benefit stopped coming for the supervised hospital sessions and second, those who began to worsen, dropped out. Although we faced a drop-out rate of 21.95%, feedback from patients suggested that they felt more cared for in the weekly supervised contacts program. Given that QSEs are a part of standard care for managing OA knee patients at our hospital, the present research project suffers from the drawback that a control arm could not be kept because of ethical issues. On the other hand, the fact that the design of our research enabled a direct comparison of neutral knees with varus-aligned knees could be considered a strength.

The statistical analysis of results within and between the groups (Table II) in the present study has generated radiological evidence to unravel the paradox as to why despite the balming effect of quadriceps strengthening exercises, which improves the pain and functionality of the knees among the patients, the knees continue to deteriorate and ultimately end up getting replaced in a sizable number of OA knees with varus. All three radiological parameters that were recorded in this study indicated that there was a structural deterioration of the knee joint under the influence of QSEs in both the neutral as well as the varus groups, with a preponderance of disease progression in knees wherein the varus deformity had already set in. This paper identifies causes for concern and sensitises the practitioners to the need for defanging the current quads strengthening methods of their deleterious effects and identifying alternate load-modifying interventions and safer regimens in medial knee OA management. Hafez *et al* have suggested that QSEs should be coupled with hamstring strengthening to normalise the altered line of action of muscular force^36^. In the present study, patients who did not get relief in pain had a mean pre-intervention varus of 9.3 whereas it was 8.75 in those for whom the VAS score improved. According to Gaasbeek, the threshold for HTO is 10° and the closer the varus angle is to this value, the more vulnerable is the knee to an increase in pain following QSEs^37^. In other words, since QSEs in this group are likely to hasten the march of disease progression to the cut-off threshold value of 10°, it is best to avoid QSEs in this group. These findings are in line with those reported by other workers^33,38^, that the treatment of medial knee OA should be tailored to the individual, and factors such as knee alignment ought to be taken into consideration. It appears prudent at the current stage of clinical evidence to stop strength training in patients who either do not benefit from QSEs or in whom the exercises cause increased pain.

A caveat is however in order. Given that our study was both short-term and involved a small number of patients, any universal extrapolation of its findings should be made with caution. There is a need for further long-term and larger studies in similar cohorts of patients to arrive at firmer conclusions. We further add that no other investigations (like MRI cartigram etc.) were performed to evaluate the amount of cartilage degeneration. All the measurements were made manually by one observer only which has the potential of bias.

## Conclusion

The present study brings to the fore the paradoxical role played by QSEs in the management of medial compartment osteoarthritis of the knee. We suggest that the staple prescription of quadriceps exercises should be avoided in varus-aligned knees.
